# Effect of membrane depolarization against *Aspergillus niger* GM31 resistant by ultra nanoclusters characterized by Ag^2+^ and Ag^3+^ oxidation state

**DOI:** 10.1038/s41598-023-29918-w

**Published:** 2023-02-15

**Authors:** Junior Bernardo Molina Hernandez, Luca Scotti, Luca Valbonetti, Luisa Gioia, Antonello Paparella, Domenico Paludi, Antonio Aceto, Maria Rosa Ciriolo, Clemencia Chaves Lopez

**Affiliations:** 1 Faculty of Bioscience and Technology for Food, Agriculture and Environment, University of Teramo, Teramo, Italy; 2 Department of Medical, Oral and Biotechnological Sciences, “G. d’Annunzio” University of Chieti-Pescara, Chieti, Italy; 3 Faculty of Veterinary Medicine, University of Teramo, Teramo, Italy; 4 Department of Biology, University of Rome “Tor Vergata”, Via della Ricerca Scientifica 1, 00133 Rome, Italy

**Keywords:** Biochemistry, Drug discovery, Microbiology

## Abstract

To date, the impossibility of treating resistant forms of bacteria and fungi (AMR) with traditional drugs is a cause for global alarm. We have made the green synthesis of Argirium silver ultra nanoclusters (Argirium-SUNCs) very effective against resistant bacteria (< 1 ppm) and mature biofilm (0.6 ppm). In vitro and preclinical tests indicate that SUNCs are approximately 10 times less toxic in human cells than bacteria. Unique chemical-physical characteristics such as particle size < 2 nm, a core composed of Ag^0^, and a shell of Ag ^+^, Ag^2+^ , Ag^3+^ never observed before in stable form in ultra pure water, explain their remarkable redox properties Otto Cars (Lancet Glob. Health 9:6, 2021). Here we show that Argirium-SUNCs have strong antimicrobial properties also against resistant *Aspergillus niger* GM31 mycelia and spore inactivation (0.6 ppm). The membrane depolarization is a primary target leading to cell death as already observed in bacteria. Being effective against both bacteria and fungi Argirium-SUNCs represent a completely different tool for the treatment of infectious diseases.

## Introduction

In the last years there has been a fast spread of multi- and pan-drug resistant bacteria (also known as “superbugs”) which are responsible of infections not treatable with existing antibiotics. Moreover, the rise of multi-drug resistance in fungi is also a cause for alarm^1,2^. Growing antimicrobial resistance (AMR) could spiral out of control of the healthcare system, according to estimates for the year 2050, deaths attributable to AMR could reach 10 million^1,3^. In addition to this, it should be considered the economic consequences due to continuous increase in health care costs. The development of new antimicrobial agents is necessary in order to overcome antimicrobial resistance. Silver nanoparticles (AgNPs) have attracted the attention of many researchers for their antibacterial and anti-fungal properties, varying between 10 and 100 ppm^4^ .The plethora of AgNPs synthesis protocols can be grouped in physical, chemical, and biological green methods^5^. Important problems have to be overcome to make the synthesis methods reproducible. The presence of reducing agents, stabilizers, and contaminants can interfere in terms of efficacy and toxicity by altering the expected results from nanoparticle formulations^5,6^. Structural characterizations are often incomplete so that, in many cases, the comparison of results is not possible^4,7^. To overcome these issues, we have recently modified an old synthesis^8^ for generate a novel silver nanoparticles, using a reproducible electrochemical method (Patent EP-18181873). Our nanoparticles exhibited antibacterial properties even against resistant strains at a very low concentration (< 1 ppm), a value much lower than that reported for other silver formulations^4^and are also very effective at deconstructing mature biofilm (0.625 ppm)^9–11^. This is because they are a novel nano material characterized by unique chemo-physical properties. Their size (< 2 nm) is the smallest of all nanoparticles so far studied, which is also why we named them Argirium Silver Ultra Nano Clusters (*Argirium-SUNCs)*^12^. All studies indicate that small nanoparticles induce greater cytotoxicity^13^ than larger ones because of the higher surface area/volume ratio that facilitates the binding around the cell membrane with its consequent damage^13^. Structural studies^12^exhibited other unique SUNCs features. The presence of metallic Ag^0^ in the core of the nanoparticle while in the external shells, due to the electron-attracting action of the water oxygen atoms, are present Ag^+^, Ag^2+^ and Ag^3+^ silver oxides never observed in a stable form before.

The consequent high anionic salvation surrounding of SUNCs (Zpuls value > − 50 mV) explains their stability for several months in ultra-pure water solution without large aggregates while the presence of silver oxides on the clusters surface explains their enhanced redox properties towards biological targets. Different mechanisms have been proposed to explain the AgNPs antimicrobial action such as oxidative stress, reduced ATP synthesis, reduced GSH concentration and enzymes inhibition^13–15^**.**

However, from the results reported in the literature^13,15^, the events leading to cell death are not always clearly distinguished from side effects/epiphenomena. We have recently identified the membrane depolarization and increased intracellular calcium level as primary events leading to bacterial death^11^. Finally, in vitro and in vivo toxicity studies reported that silver nanoparticles are no toxic against lung epithelial cells up to 100 µg/ml^16^. Also our *Argirium-SUNCs* resulted about 10 times less toxic in human cells than in bacteria and, in addition, we tested them with *Galleria mellonella* as a pre-clinical toxicity test^10^. Survival curves indicated that *Argirium-SUNCs* were no-toxic toward larvae up to the highest concentration possible used 6.8 µg/ml.

Previous studies indicated differences in anti-fungal properties of different Ag-NPs used^5,17^. In addition, in most of these studies very rarely the same nanoparticles have been tested on both bacteria and fungi. This prompted us to investigated, in addition to being antibacterial, the anti-fungal properties of *Argirium-SUNCs*.

The aim of this study was to investigate the effects of *Argirium-SUNCs* on *Aspergillus niger* , an ubiquitous fungus, which causes various diseases in different living organisms, producing mycotoxins. In humans, it causes invasive aspergillosis, particularly in immunosuppressed patients, undergoing transplants and blood cancers^18^. Several synthetic fungicides are used to combat *Aspergillus niger*, contamination in food and various environments. *Aspergillus niger*, has developed resistance to antifungal agents and is considered as “model organism” to investigate anti-fungal performance of different molecules^19^.

The results indicate that they are very effective against resistant A*spergillus niger* GM31 (0.625 ppm) and the anti-fungal mechanism was similar of SUNCs to what we have previously described in bacteria^9,10^ even if with differences related eukaryotic nature of *A. niger* GM31.

This indicates that our Ag formulation could represent a new class of molecules that overcome antimicrobial resistance. Unlike traditional antibiotics, Argirium-SUNCs being effective against bacteria and fungi represent an entirely different pharmacological strategy for dealing with infectious diseases^20^.

### Effect of SUNCs on *Aspergillus niger* GM31

**Figure 1:**
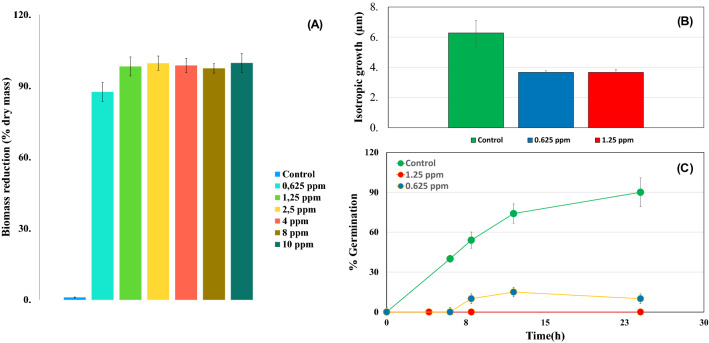
(**A**) Reduction of mycelia growth. 0,2 g of *A. niger* GM31 mycelia was growth at different concentrations of SUNCs in MEB at 28 °C for 7 days.Untreated mycelia was used as a control. The mycelial growth inhibition, measured as the percentage of dried mycelia biomass, was determined according to Eq. (1) (materials and methods). (**B**) The effect of Arg-SUNCs on the conidia germination. Conidia suspension (9.8 × 10^6^ CFU mL^−1^) were incubated at different concentrations of SUNCs at 28 °C at different time. (C) Dimension of the spores during this first phase at different concentrations of SUNCs within 4 h at 28 °C. The analysis of variance (ANOVA) was carried out to every variable studied (*P* < 0.05).

**Figure 2:**
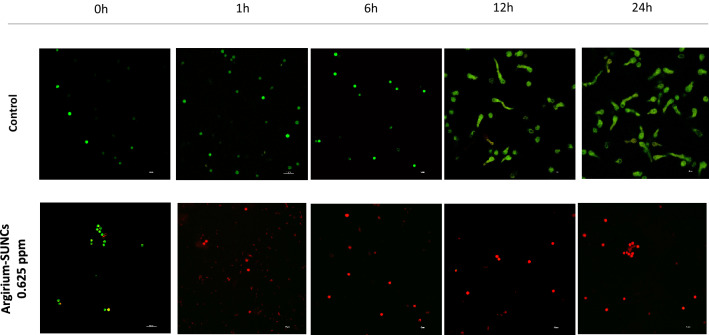
Effect of sublethal concentration (0.625 ppm) of *Argirium-SUNCs* on *A. niger* GM31 cell viability. Live and death as (green) intact fungal structure; (red) damaged fungal structure were visualized by fluorescence microscopy after staining with CFDA (carboxyfluorescein diacetate) and PI (propidium iodide). Scale bar 20 μm.

*Argirium-SUNCs* showed strong antimicrobial properties against mycelia and spore inactivation with a MIC value of 1.25 ppm (Fig. 1A,B). In particular 0.625 ppm of *Argirium-SUNCs* treatments significantly decreased the mycelia production (*P* < 0.05) of about 80% (Fig. 1A,B). With the enhancement of *Argirium-SUNCs* concentration the mycelia growth was further reduced reaching 100% already at 1.25 ppm after 7 days of incubation at 28 °C. To observe if the anti-fungal activity was fungicidal or fungistatic the treated mycelia with 1.25, 2.5, 4, 8 and 10 ppm were washed with PBS buffer and further inoculated in a new liquid media (50 mL of malt extract broth) for 7 days more at the same temperature. After this incubation period, we did not register any mycelia growth suggesting a fungicidal effect of the *Argirium-SUNCs* already at 1.25 ppm. As regard spore germination, also in this case we observed a strong reduction of germination of about 87% of the spores at 0.625 ppm in relation to the 98% of germinated spores of the control group (CTR) (Fig. 1C). Since the first morphological change in spore germination of many fungal species is the isotropic growth, in which the spore starts to swell and consequently increases its volume due to the water uptake, which is accompanied by numerous metabolic activities including respiration, RNA and protein synthesis^21,22^ , as well as trehalose breakdown^21^**,** we measured the dimension of the spores during this first phase (Fig. 1C). As evidence spores treated with *Argirium-SUNCs* delay this phase achieving only 3.66 ± 0.2 um (95% of spore) within 4 h at 28 °C (Fig. 1B and C), in contrast, with the CTR spores, which measured 6.28 ± 0.7 um (98% of spore) during the time. Figure 2 shows the cells viability. At is well-known dyes of SYTO 9 (green) and propidium iodide (PI) (red) have diverse permeability to healthful cells.

In fact, while live cells are stained with SYTO 9, dead cells with damage in cellular membrane stained with PI. As shown in Fig. 2, in CTR samples almost all cells exhibited a green fluorescence indicating that cell hyphae are alive. With the increase of *Argirium-SUNCs* concentration and exposure time, a significant reduction in green fluorescence was evidenced and the red fluorescence was markedly intensified. In particular we observed after 1 h of exposure a significant number of the spores showed a membrane disruption since PI stain the DNA of a high percentage of cells. The maximum cells killing were achieved already at 24 h after treatments (Fig. 2).

### The cell membrane depolarization, the pivot

As observed above *Argirium-SUNCs* showed a rapid anti-fungal action, thus we expected that cellular membrane was a principal target of the anti-fungal action of the nano clusters. As it is well known cells accumulate energy produced during respiration by forming both a charge gradient (membrane potential, ΔΨ) and a chemical gradient across a membrane system^23^. The ΔΨ potential is a component of a large range of essential biological functions^24^ such as ATP synthesis, flagella rotation and ammonium accumulation. We therefore studied the change in membrane potential using a fluorescent dye DiBAC4^11^ that enters into the cells when they have a depolarised membrane, binding intracellular or membrane proteins and showing green fluorescence.

**Figure 3:**
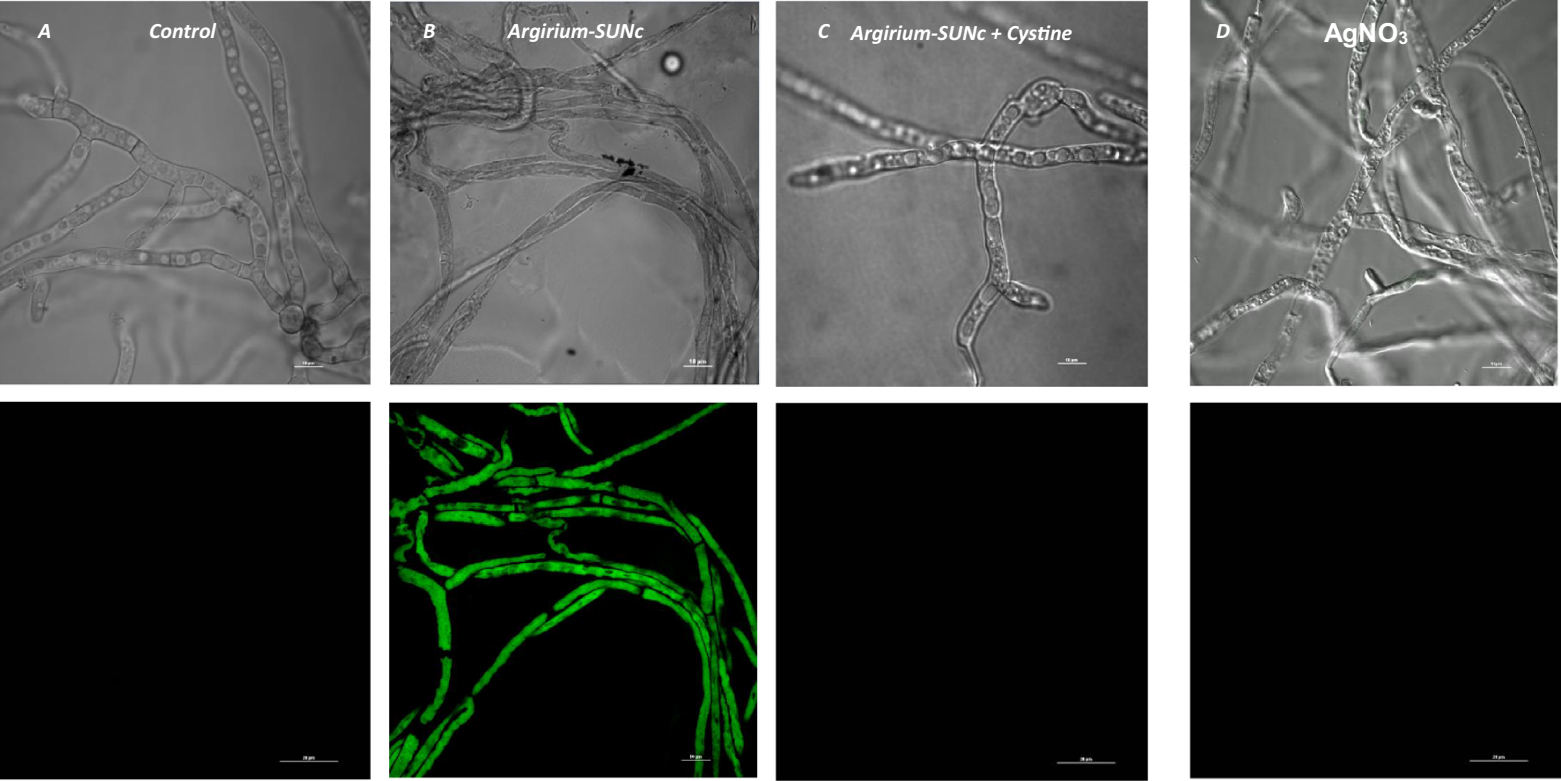
Effect of *Argirium-SUNCs* on cellular membrane depolarization. Hyphae of *A. niger* GM31 were stained with 20 μg/ml of DiBAC4 (Bis-(1,3-Dibutylbarbituric Acid Trimethine Oxonol). (**A**) Untreated hyphae; (**B**) hyphae treated with 0.625 mg L^−1^ *Argirium-SUNCs*; (**C**) Hyphae treated with *Argirium-SUNCs* + Cystine and (**D**) Hyphae treated with AgNO_3_. Scale bar of 20 μm.

As observed in Fig. 3, there is no visible fluorescence in CTR cells, indicating that they are not depolarized. In contrast, intense green fluorescence was observed in cells treated with *Argirium-SUNCs*, indicating a loss of membrane potential. We have previously observed that *Argirium-SUNCs favour* azobenzenes isomerization in aqueous solution^25^. In addition disulphides such as cystine and GSSG rapidly interact with *Argirium-SUNCs*, strongly altering their properties as indicated by the disappearance of the plasmonic spectrum (unpublished results). As shown in Fig. 3, the simultaneous addition of cystine and *Argirium-SUNCs* to treated cells (Fig. 3) inhibits fluorescence dyeing, confirming the decisive role of *Argirium-SUNCs* in the membrane depolarization process. Interestingly, the addition of Ag^+^ in the form of AgNO_3 ,_ in contrast to *Argirium-SUNCs*, does not alter the membrane potential (Fig. 3). This means that the oxidative power of Ag^+^, which is lower than that of Ag^2+^ and Ag^3+^ present in our nanoclusters, is not sufficient to depolarize the membrane. This rare oxidative states of silver were observed for the first time by works reported in^26,27^. However Ag^2+^ and Ag^3+^ species produced by anodic oxidation synthesis and stoked at − 20 °C were found to be too unstable in order to test their possible effects in any field^26^. In an other work Ag^+^/Ag^2+^/Ag^3+^ nanoparticle composites was obtained by green synthesis. This nanoparticle composition displayed an antibacterial activity in the range 0.7 and 12.5 ppm. However this nano formulation obtained from the pomegranate extract stabilized by contaminant biomolecules resulted complex and authors reported that the mechanism of dispersion and stabilization by biological macromolecules needs further study^28^.

On the contrary in ultra pure water solution our ultra nanoclusters are characterized by stable Ag^2^ + and Ag^3^ + cationic species coordinated in Ag_3_O_4_^12^ form as indicated by XRD data already deposited at the International Centre for Diffraction Data (ICDD Reference #56,443) upon request. Thus, the present results indicate for the first time that *Argirium-SUNCs* have as primary target the membrane depolarization whose loss of function leads to cell death in bacteria and fungi, both of which are characterized by a negative external membrane potential.

**Figure 4:**
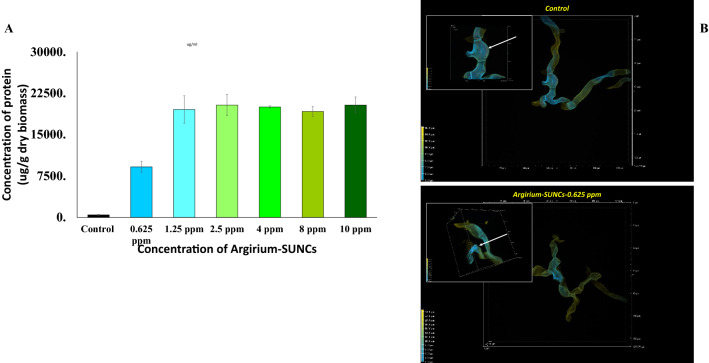
Release of intracellular proteins from *A. niger* GM31 before and after treatments with *Argirium-SUNCs*: (**A**) Protein concentration (μg/g dry biomass) in the supernatant medium after 18 h of incubation with the *Argirium-SUNCs* at 28 °C*;* (**B**) morphological change as indicate by arrow (magnification picture) after 18 h of 0.625 mg *Argirium-SUNCs* exposure at 28 °C treatments.

To the best of our knowledge, membrane depolarization is not reported as a primary effect in the case of other silver nanoparticles whose the antibacterial activity was dominated by Ag^+^ ions and by the absence of Ag^2+^ and Ag^3^ + cationic forms^5,13^. As consequence of the depolarization the membrane permeability of the fungal cells is likely compromised due to the *Argirium-SUNCs* interactions, thus we observed the morphology of the *A. niger* GM31 hyphae. As reported in Fig. 4D the CTR hyphae displayed a normal morphology with turgid cells, which coincided with the results of cell viability.

On the contrary, treated mycelia showed flattened hyphae, due to the leakage of the intracellular compounds as observed by the significant (*P* < 0.05) increase of the extracellular protein content which major values were reached at 1.25 ppm of *Argirium-SUNCs* (Fig. 4A).

The depolarization could also alter the membrane channel properties and affect increased intracellular calcium we have already observed in bacteria^29^. It has been proposed that the calcium level represents an important intracellular messenger that initiates and regulates the apoptotic process^29,30^.

## Methods and protocols

### Oxidative stress (ROS) and integrity of membrane structural component

To determine if SUNCs treatment contributes to the generation oxygen radicals (ROS) of *A. niger* GM31 mycelia, the intracellular ROS were further studied. In this case we used the dye DCFH-DA which can penetrate into the hyphae and after being hydrolysed by intracellular esterase to produce DCFH, which in presence of ROS is oxidised to DCF and emits green fluorescence.

**Figure 5:**
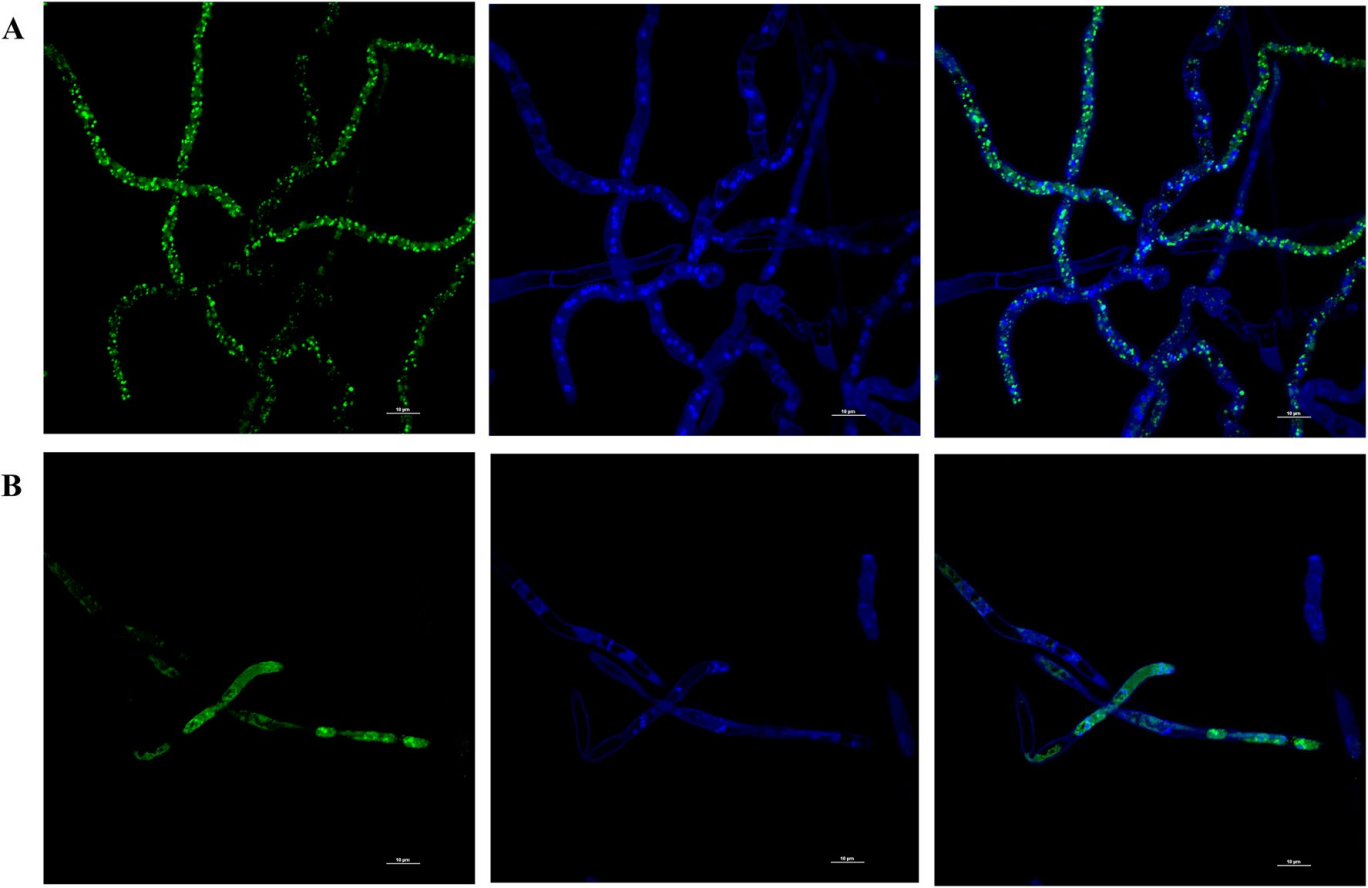
Mitochondrial staining of *A. niger* GM31 with MitoTracker Green before and after treatment with *Argirium-SUNCs*. From left to right (nucleus-mitochondrial-overlaped): fluorescence intensity of Hoechst (nucleus), MitoTracker Green (mitochondrial viability): (**A**) untreated hyphae and (**B**) treated hyphae with 0.625 mg L^−1^ of *Argirium-SUNCs*. Scale bar: 20 μm.

As illustrated in Fig. 5A, a weak fluorescence intensity was detected in CTR hyphae, indicating the normal ROS production during the aerobic metabolism of *A. niger* GM31. The fluorescence increased drastically after 1 h of *Argirium-SUNCs* exposure and almost near 75% of the hyphae showed an evident ROS accumulation (Figure. S1, b and c).

The changing pattern of the ROS-positive cells is consistent with the toxicity of metal ions like Ag°/Ag^+^ suggesting that a high concentration of ROS is a cofactor in the cellular stress of mycelia. In most of the works the Ag^+^ oxidizing effect is considered the main cause of cellular toxicity^31^. In the present and previous works we demonstrate that a greater antimicrobial effect is obtained by membrane depolarization caused by *Argirium-SUNCs* which are, at the same time, less toxic than AgNO_3_ against human cells^12^. These data, all together, confirm that the AgNO_3_ formulation and *Argirium-SUNCs* exhibit two different antimicrobial mechanisms and that our ultra-nanoclusters, although more effective against fungi and bacteria, are less toxic than AgNO_3_ against human cells^12^. We investigated if the depolarization induced by *Argirium-SUNCs* has any effects on energy metabolism, and for this purpose the mitochondrial membrane integrity was measured. It is well known that the production of energy is one of the most principal roles of the mitochondria, it also control key physiological activities, such as lipid synthesis and trafficking, aging, reactive oxygen species production, apoptosis, and cellular signaling^32^. It has been well documented that when the mitochondrial membrane is damaged, ΔΨm decreases^33^. The confocal images of CTR of the *A. niger* GM31 hyphae cells (Fig. 5A) showed an intense green fluorescence after the staining with MitoTracker dye suggesting a mitochondrial potential activity. These ΔΨm decreased upon to *Argirium-SUNCs* treatment (Fig. 5B), affecting the energy metabolism, inhibits the normal growth of *A. niger* GM31. It is well known that ΔΨm is an indicator of the integrity of the mitochondrial membrane. In a previous work it has been demonstrated that mitochondrial dysfunction participates in the induction of apoptosis as a response to some cellular damage/stress and it has been underlined how it plays a central role^34^. This pathway induces depolarization of the transmembrane potential ΔΨm, release of apoptogenic factors and loss of oxidative phosphorylation.

As the fungal cell wall is a dynamic and developmentally plastic construction, capable of compensating for the loss of β-1,3-glucan by increased chitin deposition, we investigated the accumulation of chitin and glucan after *Argirium-SUNCs* treatment through the calcofluor white probe (Figure S2). Chitin is one of the major structural components of the fungal cell wall, which plays an important role in pathogen resistance and environmental stress^3^. The microscopic examination of the chitin and glucan-stained hyphae treated with *Argirium-SUNCs* revealed an irregular deposition of chitin spots within the hyphae or spore cell wall (Figure S2 (b,c)). This could mean that irregular cell wall synthesis occurs as a adaptive response to *Argirium-SUNCs* treatments.

### Toxicity of SUNCs on mammalian cells

We have shown that treatment of the sheep fibroblasts (SAF) with SUNCs (0.625 ppm) does not affect the somatic cell's vitality. Counting the number of the live cells using Trypan blue staining there was no observed statistical difference between the treated and CTR. In addition, the absence of cytotoxic effect was confirmed using live/dead assay with Calcein-AM/PI (Fig xx).

**Figure 6:**
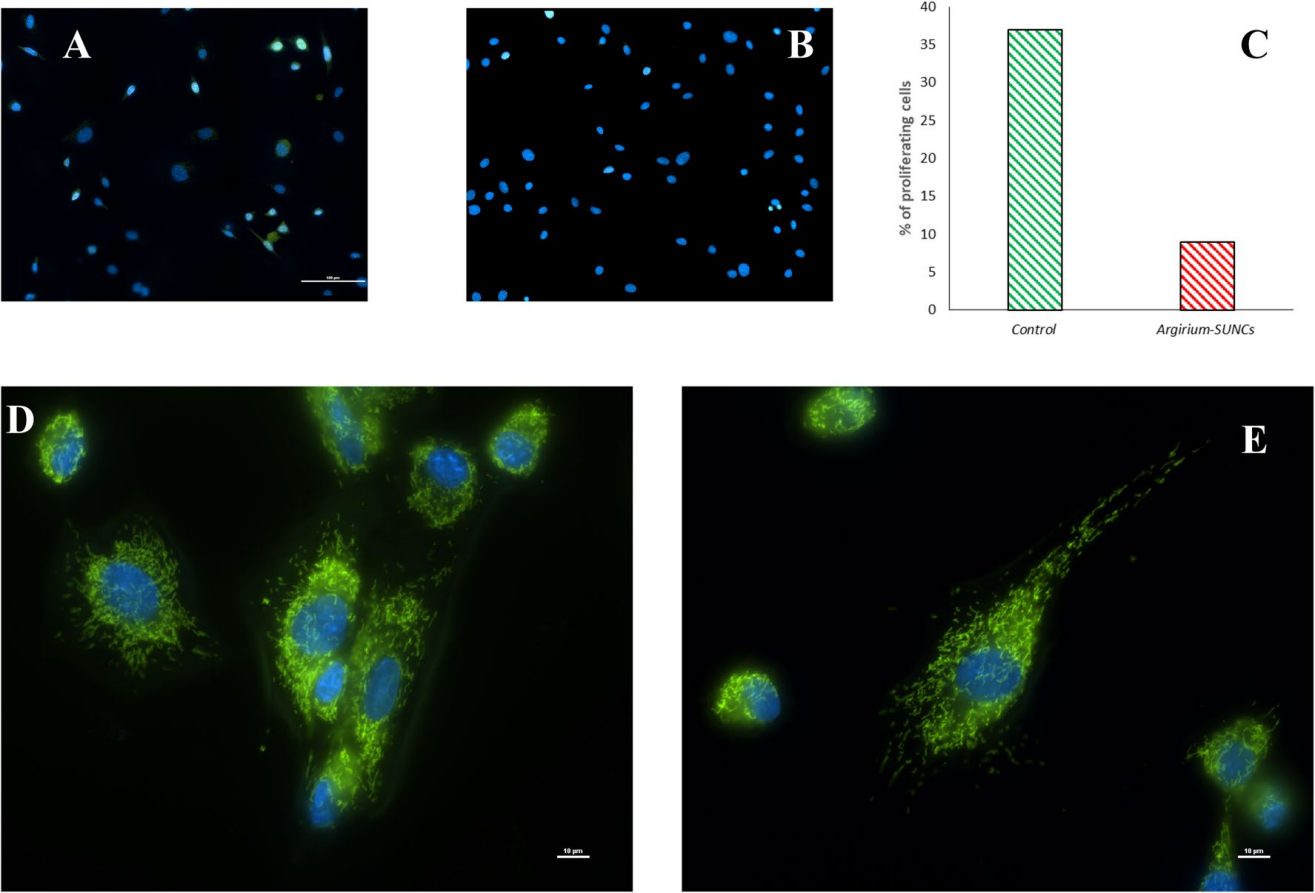
Effect of *Argirium-SUNCs* on fibroblasts. (**A**, **B**) The BrdU assay revealed that *Argirium-SUNCs* reduced cell proliferation (**B**) in SAF compared to control (**B**). The difference in the proliferating cells (%) is reported in histogram (**C**). The images show BrdU positive cells (green), indicative of replicating cells. All nuclei were counterstained with Hoechst 33342 (blue). Merge means Hoechst + BrdU (**A**, **B**) (n = 3 images) (*p < 0.0005; **C**) . Scale bar = 100 μm; magnification is 20X (**D**, **E**) Sheep fibroblasts, that were previously subjected to *Arg-SUNCs* treatment for 24 h (**D**) and control non-treated (**E**), were stained with Mitotracker green dye. Pictures show the amount and localisation of the active mitochondria. No observed important reductions in dye accumulation between the treated *Argirium-SUNCs* and CTR group indicating that SUNCs does not affect mitochondrial functionality; nuclei were stained with Hoechst 33342; Experiments were conducted in triplicate. Scale bars: 10 μm.

We assessed the functionality of mitochondria using the Mito-Tracker Green dye. There were no observed important reductions in dye accumulation between the treated *Argirium-SUNCs* and CTR group (Fig. 6D,E). Treated SAF displayed only a lower proliferation rate compared to the CTR group, as shown by the significant difference in the percentage of BrdU-positive cells between SAF exposed to *Argirium-SUNCs* for 24 h compared to the CTR group (9% vs. 35%, respectively; *p* < 0.001) (Fig. 6A–C).

These results indicate that SAFs are more resistant than *A. niger* GM31 to SUNCs.This is in agreement with our previous toxicity studies in which the MTT values of mammalian cells (HEK-293, HaCaT, HMEC) were always higher^9,10,12^ than the MIC values observed in bacteria. A preclinical toxicity model also indicated that *Argirium-SUNCs* was not toxic to *Galleria mellonella* larvae up to the highest concentration of 6.8 ppm that could be used. The presence of metal ion scavenger systems such as metallothionein and/or the positive external potential of the mammalian membrane could be some of the characteristic properties of human cells that explain their greater resistance to *Argirium-SUNCs* than bacteria and fungi.

### Synthesis and characterization of Arg-SUNCs

**Figure 7:**
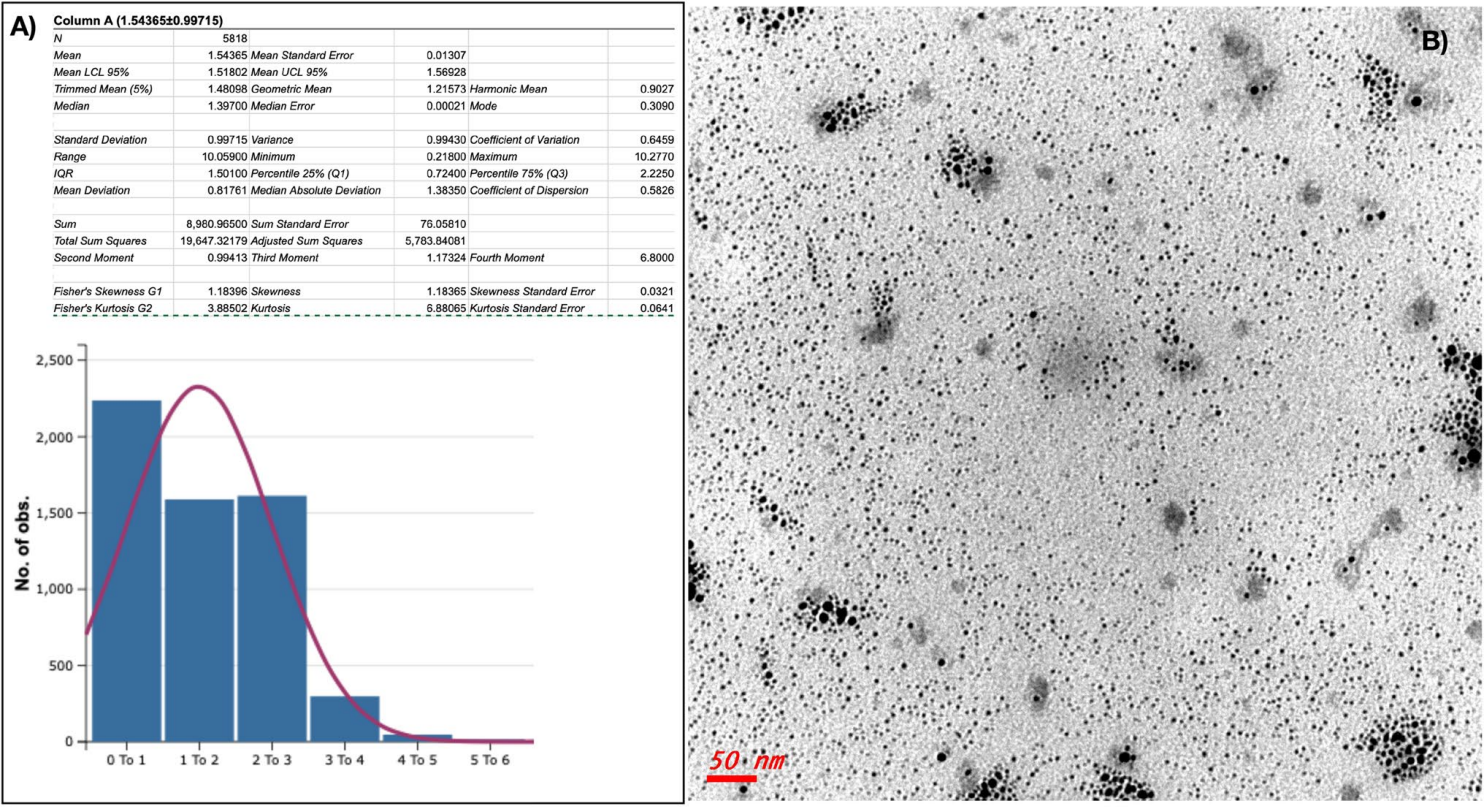
(**A**) TEM and (**B**) Statistical Distribution of Argirum SUNc ultra nanoclusters, Software: StatPlus-2 Mac,Version v8. IMAGEJ-2 Version 1.53t.

ARGIRIUM-SUNCs (Arg-SUNCs) were synthesized with a reproducible method at 20–40 ppm water solution concentration as measured by Ionic selective Electrode (ISE) and TEM (Fig. 7) in ultra-pure water without stabilizing agents or other chemical components as previously reported^12^. The method is protected by European Patent (EP-18181873.3) and trade mark (Argirium SUNCs ™).

The main chemical–physical properties (Table 1) of Arg-SUNCs as determined by TEM , XR-SEM, X-Ray Diffraction (XRD) , X-ray photoelectron spectroscopy (XPS) , MALDI TOF, Dynamic light scattering (DLS), UV–Vis, ISE are : a very small size 1.79 nm ± 1.004 (ultra nano clusters), plasmonic resonance spectrum (λ max at 410 nm) and non-spherical shape^11^.

**Table 1:** Chemical–physical properties of Argirium SUNCs. Are present collected data of this manuscript and precedents for a detailed description.

Chemical physical-characteristics	pH	Size (nm)	SEM-XRD data	Oxidative state		Zpuls	Temperature (C°)	ORP
Argirum SUNcs	7.8	1.54 ± 0.013	97.67 / 13.84	374.53 and 373.32 eV	368.53 and 367.32 eV	− 40/− 70 mV	20/80	+ 400 mV
*Solvent solutions: UPW (Ultra Pure Water)*	*Stability range 3–12*	*Mean* ± *DS*	*Ag / O*	*Ag 3d3/2*	*Ag 3d5/2*	electrochemical equilibrium at the particle-liquid interface	*Stability range*	Oxidative and Reduction Potential
Data reported in references number 1,8								
In Argirium SUNCs solutions are not present stabilizants and any organic or inorganic compounds. Elementary composition: Ag (99,9% ) and water. Stock solutions are prepared at different concentrations (1—40 ppm)								

The X-ray diffraction and XPS analysis revealed the presence of Ag_3_O_4_ and AgO crystalline phase corresponding to Ag ^+^, Ag^2+^, Ag^3+^ silver oxides on the surface of the Arg-SUNCs and Ag° in the core. As a consequence SUNCs are characterized by negative solvation shell (Zeta Potential values in the range − 40/ − 70 mV) (Figure S3). The high absolute zeta potential value suggests that nanoparticles surrounded by anionic salvation tend to repulse each other, avoiding any aggregation process and explains the long stability (> 1 year). The Oxidation–reduction potential (ORP) is resulted + 400 mV in comparison to − 90 mV obtained from the Dithiothreitol (DTT) Reducing Agent standard solution (Thermo Scientific™).

This surprisingly high ORP value indicates an high oxidative capacity above the Ag ° torque (+ 0.80 Volt)//H_2_O_2_ (+ 1.78 Volt) as deduced from the redox values of standard potentials^35^and compatible with an unusual superficial presence of Ag^2+^, Ag^3+^ was never observed before in stable form in ultra-pure water.

## Result and discussion

### Antifungal activity

#### Strain and inoculum

In this study we used the strain *A. niger* GM31 provided by the Faculty of Bioscience and Technology for Food, Agriculture and Environment, University of Teramo. The strain was isolated previously from food sources and identify by molecular methods by using the internal transcribed spacer (ITS) region. Primers used were ITS1/ITS4 as described by Molina Henandez et al.^11^.

The strain from stock culture tubes was reactivated and cultivated in malt extract agar (MEA-OXOID) medium plates and incubated for 5 days at 28 °C, in the dark. Conidia formed were collected in 1 mL of sterile physiological solution and washed twice. Successively the concentration of the spores was adjusted to 10^5^/ml and 100μL of conidia suspension were inoculated in 250 mL of MEA and incubated for 5 days, at 28 °C in a rotary shaker (110 g) incubator (Centomat BS-T, B.Braun, Milan, Italy). Successively fungal mycelium was collected by centrifugation (15 min, 4 °C, 100 g), and used for further experiments.

#### Determination of the antifungal activity of NANO

*F*ollowing this equation:

1 $${\text{Y}}\left( {\text{t}} \right) = {\text{b}}1 \times \exp \left( { - \exp \left( {\left( {{\text{b}}2 \times 2.7182/{\text{b}}1} \right) \times \left( {{\text{b}}3 - t} \right) + 1} \right)} \right)$$

where, the dried mass **t** indicates the dried mycelia biomass after a determined incubation time (t), while the dried mass control indicates the dried mycelial biomass of the untreated sample (at the same time of incubation).

The minimal inhibitory concentrations (MIC) of SUNCs against the *A. niger* GM31 mycelia was determined in flasks of 100 ml containing 40 ml of malt extract broth (MEB) in which 0.625, 1.25, 2.5, 4, 8 and 10 mg L^−1^ of *Argirium-SUNCs* previously filtered (0.22 μm pore filter -Whatman International, Maidstone, UK) were added, successively 0.20 g of mycelium were inoculated and incubated at 28 °C in a rotary shaker (110 g). Untreated mycelia were used as a control. Three different replicates for each concentration were performed and the experiment was repeated two times. The mycelial growth inhibition, measured as the percentage of dried mycelia biomass, was determined according to Scroccarello et al.^19^**.**

The effect of *Argirium-SUNCs* on the conidia germination was evaluated following the methodology reported by Peralta-Ruiz et al.^36^. Briefly, twenty microliters of conidia suspension (9.8 × 10^6^ CFU mL ^−1^) were deposited onto slides previously coated with a thin layer of MEA medium, and immediately added with the different concentration of Arg-SUNCs. The slides were incubated at 28 °C and observed with a phase-contrast optical microscope after 4, 6, 8, 10 and 24 h of treatment. Conidia were considered germinated when the germinative tube resulted longer than the same conidia.

#### Impact of Arg-SUNCs on fungal membrane depolarization

Cytoplasmic membrane depolarization of *Aspergillus nigerMG13* was measured using a membrane potential sensitive probe DiBAC_4_ (Bis-(1,3-Dibutylbarbituric Acid Trimethine Oxonol, Invitrogen). Dye uptake and resultant self-quenching are modulated by the membrane potential; thus the samples were prepared by putting 100 μL of conida solution standardized in 1 ml MEB growing medium on a sterile coverslip (22 mm × 22 mm), incubated at 28 °C for 18 h. Then, one aliquoted equivalent of sub MIC concentration (0.625 mg L^−1^ Arg-SUNCs) were added onto coverslips and incubated for 6 h, the control was prepared by adding 100 μL of Milli-Q water. Subsequently, coverslips were washed with PBS and the adherent hyphae were treated with an equivalent volume of 20 μg/ml of DiBAC_4_ and incubated for 30 min in the dark at room temperature and finally washed twice with PBS. Stained *Aspergillus niger* GM31 hyphae were examined and the decrease in potential was monitored by the increase in fluorescence under a fluorescence microscope at 493 nm excitation and 516 nm emission wavelengths. Measurements were repeated at least twice.

#### Intracellular ROS accumulation

To evaluate the intracellular ROS accumulation after *Argirium-SUNCs* treatment, we used also sublethal concentration (0.625 mg L^−1^ Arg-SUNCs) of the nano material. The ROS detection was assessed using 2´,7´- dichloro dichlorofluorescein diacetate (H_2_DCFDA; Molecular Probes®) described by Lui et al.^37^ with some modification. Condia were cultured in MEB medium and treated as described above. After treatments with *Argirium-SUNCs*, the adherent hyphae were washed with phosphate buffer saline (PBS- pH 7). Then 10 µM H_2_DCFDA (dissolved in dimethyl sulfoxide) was added and incubated for 1 h and washed twice with PBS. For fluorescence detection was measured at excitation and emission wavelengths of 485 nm and 535 nm.

#### Chitin accumulation

For detection of Chitin accumulation, germinated conidia were stained with calcofluor white and Blue Evans (Sigma), according to the methodology reported by Geißel et al. 2018 with some modification. After 18 h of incubation hyphae were treated with 0.625 mg L^-1^ of Arg-SUNCs for 18 h, immediately remove supernatant, and resuspended in MEB for 14 h of incubation. Samples were stained with 1 ml (10 mg ml^−1^) calcofluor white dissolved in Evans Blue for approximately 1 min. For fluorescence detection was measured at excitation and emission wavelengths of 423–443 nm.

#### Changes in mitochondrial activity

To detect if *Argirium-SUNCs* induce a change of mitochondrial and nuclei, hyphae were stained with the fluorescent dye MitoTracker (MT) Green FM (Invitrogen) and Hoechst 33,342 (Sigma) according to the manufacturer’s protocol. Also, in this case we used 0.625 mg L^−1^ *Arg-SUNCs*. The procedure was modified for *Aspergillus niger* staining. Spores were cultured in MEB medium and treated as described above. After of incubation period, hyphae were stained with MT green at 37 °C /45 min, the medium was removed and immediately counterstained with Hoechst 33342 and incubated at 25 °C/for 10 min. Finally, fluorescence was measured.

#### Release intracellular proteins

The release of intracellular protein after treatment with Argirium-SUNCs was estimated using the method of Bradford. 10 ul of supernatant (MEB) was used for measuring the protein contents and analyzed using UV spectrophotometer (Jenway™ Spectrophotometry UV–Visible Jenway™ 6305, Dunmow, EU). For quantitative analysis, bovine serum albumin (BSA) was used as a standard protein. Data was statistical analyzed for five repetitions using ANOVA (*p* ≤ 0.05; Tukey HSD post-hoc test).

### Cell toxicity

#### Cell culture and treatments

Sheep adult fibroblasts (SAF) used for the experiments have been derived from one month-old sheep, previously collected from a local slaughterhouse as refuse animal material. Primary cultures of SAF were obtained from small pieces of ears by mechanical (by blades) and enzymatic (by trypsin digestion at 38.5 °C) disaggregation. Cells were expanded in Minimum Essential Medium (MEM) enriched with 10% Fetal Bovine Serum (FBS), 2 mM l-Glutamine, 26 mM NaHCO_3_ and 50 μg/ml Gentamicin. After three passages, cells were assigned to the following groups: (i) treated group (NP), cultured in the above medium supplemented with 0.625 ppm of *Arg-SUNCs* for 24 h; (ii) control group (CTR), cultured in medium without NP. The working dose of the *Argirium-SUNCs* tested on SAF in this study (0.625 ppm) was chosen on the basis of preliminary experiments carried out to assess the NP concentration that produced significant effects on fungal cells. Cell vitality was evaluated using Trypan blue staining.

**Figure 8:**
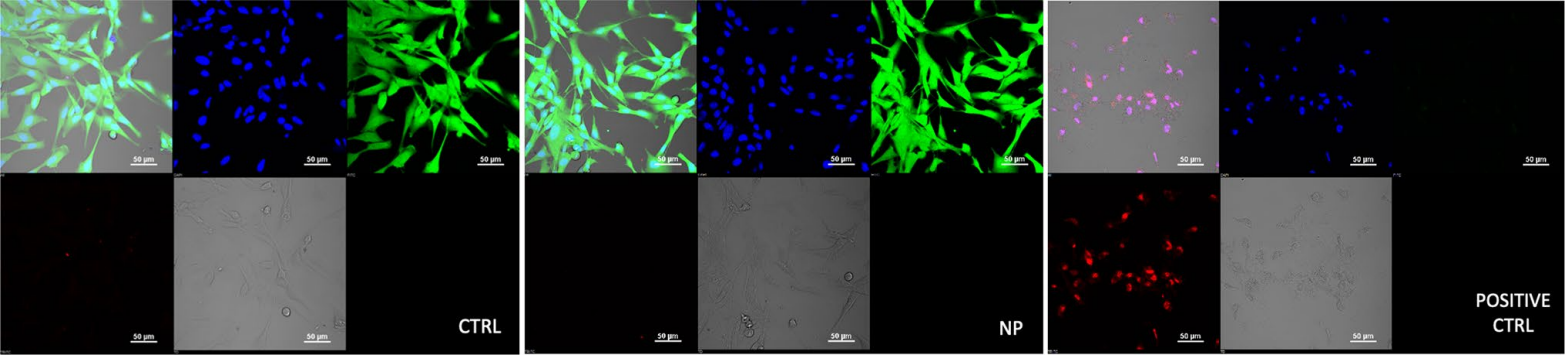
LIVE/DEAD assay in SAF cultured for 24 h with or without NP 0,625 ppm. A cell viability assay was used with SAF cultures for simultaneous fluorescence staining of viable cells (Calcein-AM, green fluorescence) and dead cells (Propidium Iodide PI, red fluorescence). The total cell number was evaluated using the viable nucleic acid staining Hoechst 33,342 (blue fluorescence). Confocal microscopy was used for image acquisition. As shown in images, both in CTRL and in NP 0,625 ppm groups, cells emit a bright green fluorescence. On the contrary, in POSITIVE CTRL, cells did not show any green fluorescence while nuclei emit red fluorescence. **CTRL:** SAF fibroblast cultured for 24 h without NP; **NP:** SAF cultured for 24 h with NP at the concentration of 0,625 ppm; **POSITIVE CTRL:** a sample of SAF cultured for 24 h in presence of very high/cytotoxic concentration (12,5 ppm) of NP was used as a positive control.

In addition to evaluate the cytotoxic effect in SAF cultured for 24 h in presence of NP 0.625 ppm, a LIVE/DEAD assay (Calcein AM/Propidium Iodide, PI, Invitrogen) was used too (Fig. 8). This cell viability assay allows the simultaneous fluorescence staining of viable cells (1 uM Calcein-AM for 20 min, green fluorescence) and dead cells (5 μM Propidium Iodide/PI for 5 min, red fluorescence). Calcein-AM fluoresces green, relying on esterase activity present only in metabolically active viable cells. PI is a polar nucleic acid dye that is excluded by the membrane of live cells, but enters the damaged membrane of dead cells and emits nuclear red fluorescence.

The total cell number was evaluated using the viable nucleic acid staining Hoechst 33342 (1 ug/ml for 5 min, blue fluorescence). SAF cultured for 24 h in presence of very high/cytotoxic concentration (12.5 ppm) of NP were used as a positive control. Images were acquired by confocal microscopy at 20X magnification, using appropriate filter sets for the three dyes.

#### Cell proliferation assay

Cell proliferation was evaluated by an immunocytochemistry assay for 5-bromo-2′-deoxyuridine (BrdU), a thymidine analog incorporated during S-phase in replicating cells if previously added to culture medium. One day before the immunocytochemistry, cells were plated in multiwell slides (Millicell EZ Slide, Millipore), in number of 10,000/well. Briefly, 24 h post treatment with *Argirium-SUNCs*, SAF were incubated with 100 μM BrdU for 4–6 h, fixed in cold methanol for 20 min and permeabilized with 0.1% Triton X-100, 15 min at room temperature (RT). Next, cells were treated with 4 N HCl 30 min, RT, and incubated with primary antibody (Ab I) (mouse anti-BrdU, monoclonal antibody, B2531, Sigma) 1:100 in blocking solution (BS) (0.1% Bovine Serum Albumin (BSA) in PBS) at 4 °C, overnight. Then, cells were incubated with secondary antibody (Ab II) (rabbit anti-mouse IgG-FITC polyclonal antibody, F9137, Sigma) 1:500 in BS for 2 h RT, and counterstained with 0.5 μg/ml Hoechst for 5 min. Between all steps, cells were washed twice in PBS, 5 min, RT. Finally, slides were mounted with Fluoromount and observed under an epifluorescent microscope Nikon Eclipse E600, at 20 × magnification. All nuclei were counted, while only the BrdU-positive nuclei have been considered as replicating cells. Cell proliferation rate was obtained by the ratio between the number of BrdU-positive cells (green) and the total number of cells (blue). For statistical valence, we considered at least 200 nuclei for each group.

## Materials and methods

### MitoTracker assay

To assess mitochondrial functionality, SAF cells after incubation with NP were further incubated at 38.5 °C for 30 min in medium supplemented with 0.5 mM MitoTracker green (Invitrogen, Milan, Italy), a cell permeant fluorescent probe that accumulates in active mitochondria. Then, cells were counterstained with 0.5 μg/ml Hoechst for 5 min, washed in PBS and mounted on glass slides. The intensity of the fluorescent signal indicating mitochondrial activity was measured using the confocal microscope Nikon Eclipse E600.

### Statistical analysis

The analysis of variance (ANOVA) was carried out to every variable studied and Tukey post-hoc test (HSD) was performed to analyze the statistical difference among means. Principal Components Analysis (PCA) based on Spearman correlation matrix, was obtained using all the variables to reduce dimensionality and better represent the whole dataset. Data were analyzed using both STATISTICA software (StatSoft, Hamburg, Germany) and XLSTAT 2021 software (Addinsoft, Paris, France).

### Inactivation of mycelia and spore germination

*3.4 Argirium-SUNCs* showed strong antimicrobial properties against mycelia and spore Inactivation MIC_S_ values of 1.25 ppm (Fig. 1A). In particular 0.625 ppm of *Argirium-SUNCs* treatments significantly decreased the mycelia production (*P* < 0.05) of about 80%. With the enhancement of *Arg-SUNCs* concentration the mycelia growth was further reduced reaching 100% already at 1.25 ppm after 7 days of incubation at 28 °C. To observe if the anti-fungal activity was fungicidal of fungistatic the treated mycelia with 1.25, 2.5, 4, 8 and 10 ppm were washed with PBS buffer and further inoculated in a new liquid media for 7 days more at the same temperature. After these incubation period, we did not register any mycelia growth suggesting a fungicidal effect of the *Arg-SUNCs* already at 1.25 ppm.

## Conclusions

The presence of stable Ag^2+^ and Ag^3+^ cationic forms in ultra pure water solution is a novelty that distinguishes *Argirium-SUNCs* from any other nanomaterial. The higher redox capacities of our ultra-nano clusters compared to Ag^+^ explain their antibacterial and anti-fungal efficacy at a very low concentration, as well as their unique antimicrobial mechanism. The main biological target of *Argirium-SUNCs* is the cell membrane whose depolarization and subsequent loss of function leads to bacterial and fungal death. Not surprisingly, the same mechanism is not reported in other silver formulations usually characterized by Ag^0^ and Ag^+^ elements which we have found to be incapable of depolarizing the cell membrane. In vitro and preclinical toxicity studies indicate that *Argirium-SUNCs* are about 10 times less toxic in human cells than in bacteria and fungi.

The specific antibacterial mechanism and experimental evidence indicate that *Argirium-SUNCs* overcome the phenomenon of the antibiotic resistance. Unlike traditional drugs, being effective against both bacteria and fungi, they represent a new and completely different pharmacological strategy against infectious diseases.

## Supplementary Information

Supplementary Information 1.

Supplementary Information 2.

## Data Availability

The datasets used and/or analyzed during the current study are available from the corresponding author. The patent referenced in the manuscript is held only by Luca Scotti without any potential conflict of interest.
