# Supplementary material for: Effect of membrane depolarization against Aspergillus niger GM31 resistant by ultra nanoclusters characterized by Ag2+ and Ag3+ oxidation state
Source: Sci Rep. 2023 Feb 15;13:2716. doi: 10.1038/s41598-023-29918-w (PMC9932144; doi:10.1038/s41598-023-29918-w)
Supplement: Supplementary file 1 — Supplementary Information 1. [file 41598_2023_29918_MOESM1_ESM.pptx]

## Slide 1
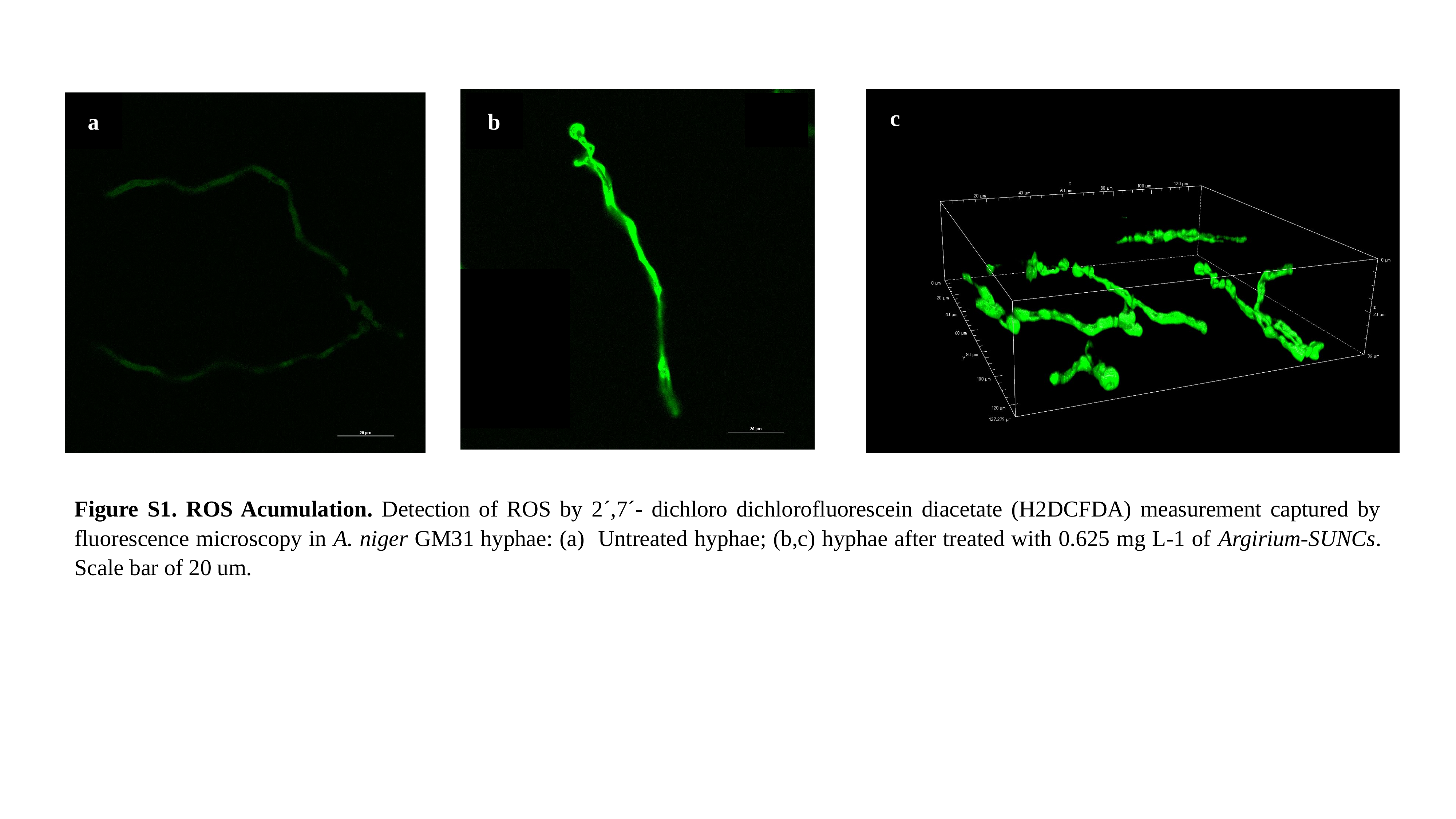

c
a
b
Figure S1. ROS Acumulation. Detection of ROS by 2´,7´- dichloro dichlorofluorescein diacetate (H2DCFDA) measurement captured by fluorescence microscopy in A. niger GM31 hyphae: (a) Untreated hyphae; (b,c) hyphae after treated with 0.625 mg L-1 of Argirium-SUNCs. Scale bar of 20 um.

## Slide 2
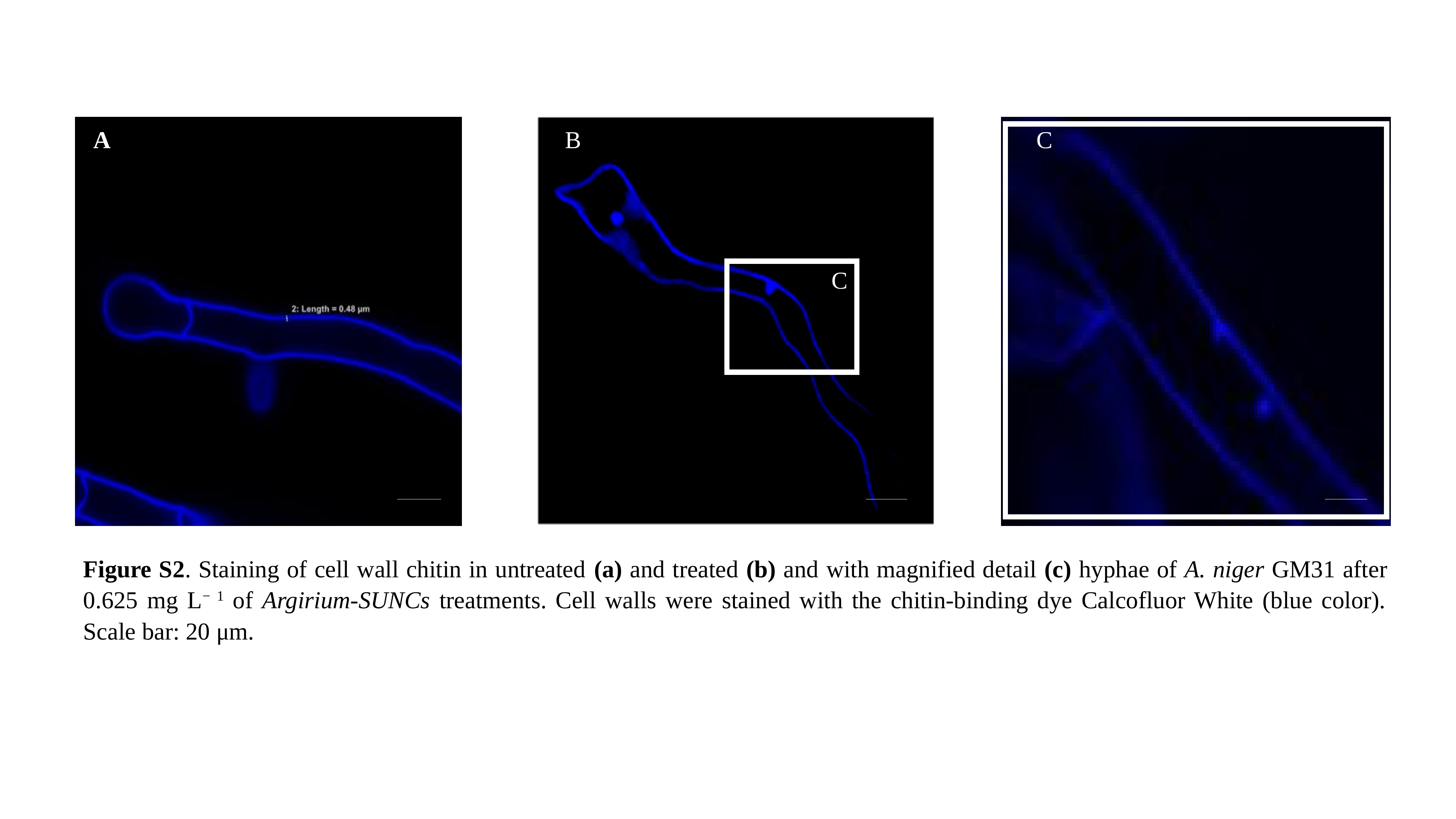

A
C
B
C
Figure S2. Staining of cell wall chitin in untreated (a) and treated (b) and with magnified detail (c) hyphae of A. niger GM31 after 0.625 mg L− 1 of Argirium-SUNCs treatments. Cell walls were stained with the chitin-binding dye Calcofluor White (blue color). Scale bar: 20 μm.
