# Supplementary material for: Effect of membrane depolarization against Aspergillus niger GM31 resistant by ultra nanoclusters characterized by Ag2+ and Ag3+ oxidation state
Source: Sci Rep. 2023 Feb 15;13:2716. doi: 10.1038/s41598-023-29918-w (PMC9932144; doi:10.1038/s41598-023-29918-w)
Supplement: Supplementary file 2 — Supplementary Information 2. [file 41598_2023_29918_MOESM2_ESM.pptx]

## Slide 1
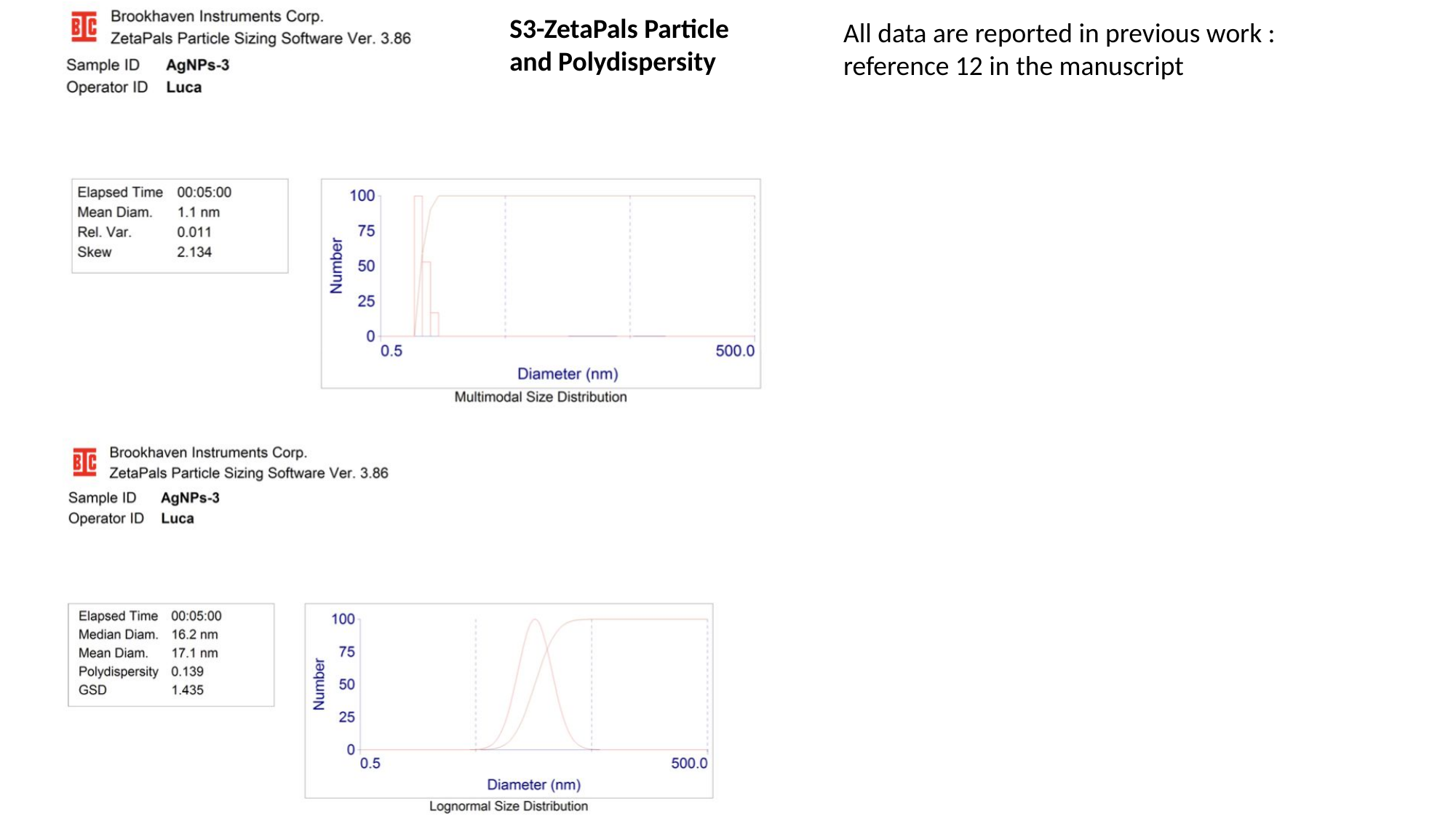

S3-ZetaPals Particle and Polydispersity
All data are reported in previous work : reference 12 in the manuscript

## Slide 2
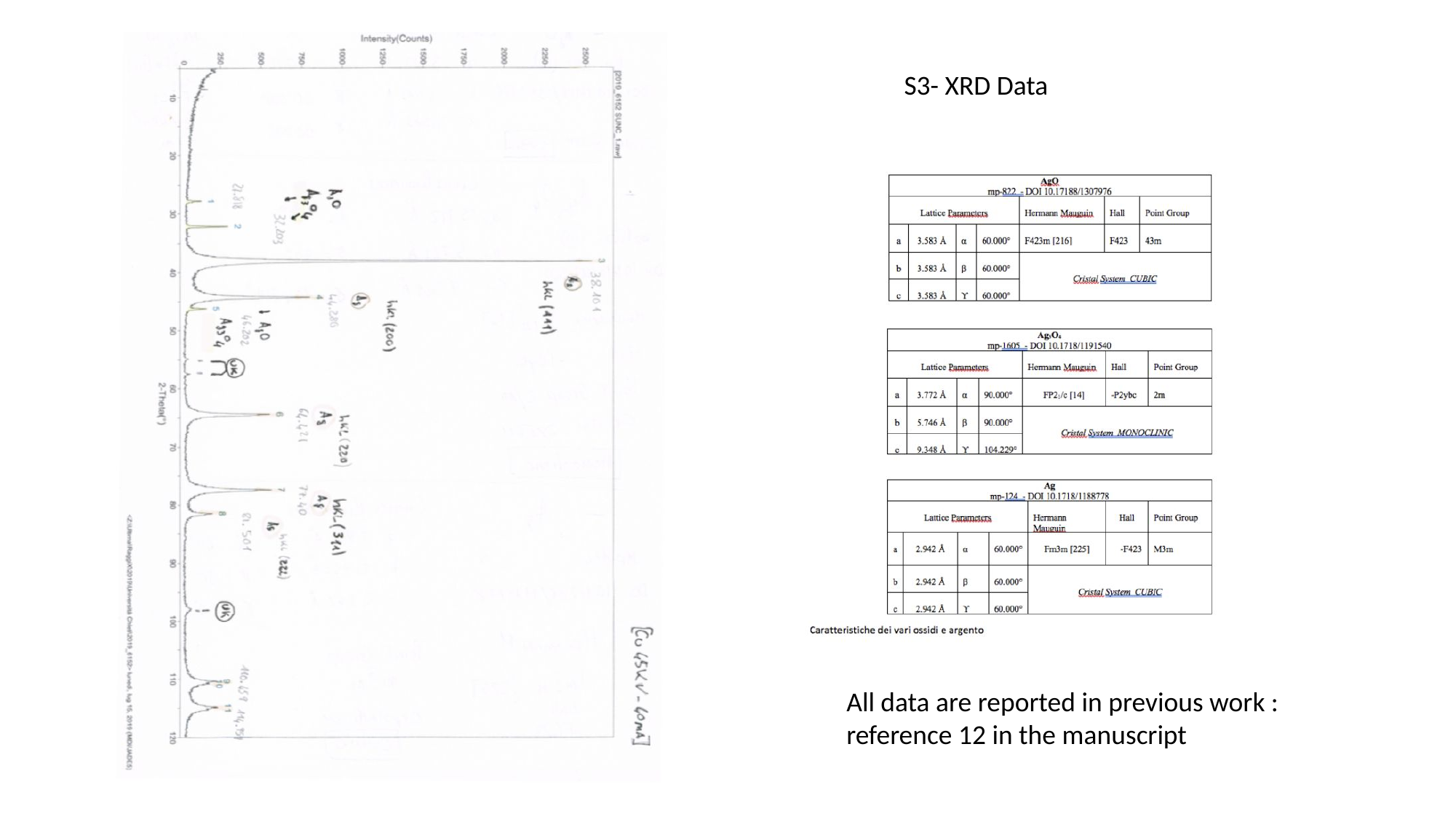

S3- XRD Data
All data are reported in previous work : reference 12 in the manuscript
